# Involvement of the systemic microcirculation in pediatric uveitis

**DOI:** 10.1186/s12969-023-00896-7

**Published:** 2023-10-02

**Authors:** Carlyn V. Kouwenberg, Julia Spierings, Evianne L. de Groot, Joke H. de Boer, Viera Kalinina Ayuso

**Affiliations:** 1 Department of Ophthalmology, University Medical Center Utrecht, Heidelberglaan 100, Utrecht, 3584 CX The Netherlands; 2 Department of Rheumatology & Clinical Immunology, University Medical Center Utrecht, Utrecht, the Netherlands

**Keywords:** Pediatric uveitis, Microcirculation, Nailfold capillaroscopy

## Abstract

**Background:**

Pediatric uveitis is a severe inflammatory ocular condition that can lead to sight-threatening complications and can negatively impact quality of life. The retinal microcirculation is often affected in intermediate uveitis and panuveitis. Here, we examined the extraocular (i.e., systemic) microcirculation in pediatric uveitis cases and healthy controls using nailfold capillaroscopy (NFC).

**Methods:**

We performed NFC in 119 children with noninfectious uveitis and 25 healthy pediatric controls, and assessed the following parameters: capillary density (number of capillaries/mm), dilated capillaries (apex > 20 µm), avascular area, the presence of microhemorrhages, and capillary morphology. Differences in NFC parameters between cases and controls were calculated using regression analysis after adjusting for age and sex.

**Results:**

The mean (± SD) age of the patient group was 13.7 (± 3) years, with 56% females; 46%, 18%, and 36% of cases presented as anterior uveitis, intermediate uveitis, and panuveitis, respectively, with an overall mean disease duration of 4.7 (± 4.0) years. Compared to the control group, the pediatric uveitis cases had a significantly higher number of dilated capillaries/mm and a higher prevalence of ramified capillaries. Moreover, compared to the control group the intermediate uveitis cases had a significantly higher number of dilated capillaries, whereas the anterior uveitis cases had a lower capillary density and a higher prevalence of ramified capillaries.

**Conclusions:**

Children with uveitis without systemic disease can present with changes in systemic microcirculation. These changes vary amongst the subtypes of uveitis.

**Supplementary Information:**

The online version contains supplementary material available at 10.1186/s12969-023-00896-7.

## Background

Uveitis is a complex, potentially sight-threatening ocular condition characterized by inflammation of the uvea and nearby structures involving the vessels in the retina and sclera [1, 2]. Vessels in the anterior uveal tract may be affected as well, visible as anterior chamber flare caused by the breakdown of the blood-aqueous barrier due to the inflammatory process. Pediatric uveitis can develop in conjunction with a systemic condition, of which juvenile idiopathic arthritis (JIA) is the most common and best-studied. In up to 60% of cases, the etiology remains unknown, and these cases are considered an isolated idiopathic inflammatory eye condition [1, 3–6]. Inflammatory involvement of the ocular vasculature is common in patients with intermediate uveitis or panuveitis and can include retinal vasculitis, diffuse retinal capillary leakage, and/or macular edema [7–10]. However, whether changes in the extraocular vascular and/or microcirculation are involved in pediatric patients with uveitis is currently unknown.

Nailfold capillaroscopy (NFC) is a noninvasive diagnostic technique used to evaluate the microcirculation in the nailfold. This technique is commonly used in the medical fields of rheumatology and dermatology, revealing various abnormal patterns associated with diseases such as systemic sclerosis and systemic lupus erythematosus (SLE) [11–14]. Ocular manifestations, including changes in the retinal or choroidal microcirculation, are described in these rheumatic diseases. More recently, NFC has also been used to detect microvascular abnormalities in ocular diseases, particularly glaucoma [15–17]. Moreover, Chen et al*.* used NFC in adults with uveitis and found abnormalities in the systemic microcirculation that were correlated with peripheral retinal leakage [18]. Because NFC is noninvasive, easy to perform, and relatively rapid, it is highly suitable for use in children [14–17]. To date, however, the use of NFC in children with uveitis has not been described. Therefore, the primary aim of this study was to compare NFC findings between pediatric uveitis cases and healthy pediatric controls, and to compare findings between uveitis subtypes. Our results may provide new insights into the pathogenesis of this potentially sight-threatening pediatric condition, as well as new insights regarding the development and application of new diagnostic and/or prognostic biomarkers.

## Methods

### Study population

The participants in this cross-sectional study were recruited between June 2020 and June 2022 at the Ophthalmology Department of the University Medical Center Utrecht (UMCU), the Netherlands, a tertiary referral center. The pediatric control group consisted of children with strabismus and no evidence of a concomitant inflammatory condition. We excluded participants with any cardiovascular or other systemic disease not associated with uveitis, including type 2 diabetes mellitus and hypertension. We also excluded participants with infectious uveitis based on serology or ocular fluid analysis. All cases were diagnosed by a pediatric uveitis specialist at the UMCU Department of Ophthalmology according to the SUN criteria [2]. The presence of associated systemic diseases was assessed in accordance with current diagnostic criteria for pediatric uveitis, and all patients were assessed by pediatricians and/or pediatric rheumatologists [19]. The analytical screening performed prior to classifying uveitis as idiopathic is shown in Additional File 1. All participants and/or their legal guardian provided written informed consent before participating in this study. This study adhered to the tenets of the Declaration of Helsinki and was approved by the UMCU Medical Research Ethics Committee (protocol number: 20–317).

### Clinical data

Demographic data obtained from all participants included age at NFC assessment and sex; in addition, the following disease-specific information was collected for the patients with uveitis: date of uveitis diagnosis, uveitis anatomic subtype (anterior uveitis, intermediate uveitis, or panuveitis), uveitis disease duration, presence (and type, if applicable) of an associated systemic disease, and laboratory results of antinuclear antibody (ANA) testing. ANA positivity was defined as a titer of ≥ 1:160 detected on the HEp-2 indirect immunofluorescence assay on 2 occasions at least 3 months apart [20]. The following ophthalmological findings were reported: presence of anterior chamber flare of 1 + or higher, presence of cystoid macular edema (CME), papillitis, retinal vasculitis and/or retinal capillary leakage on fluorescein angiography (FA). CME was defined as the presence of macular thickening with cyst formation visible on macular OCT and/or FA [21–23]. Papillitis was defined as the presence of optic disc hyperfluorescence and/or leakage on FA, inflammatory optic disc swelling, and/or retinal nerve fiber layer thickness of > 130 µm on OCT while excluding all other noninflammatory causes of optic disc swelling [24]. In order to monitor changes in NFC findings over time, a small prospective cohort consisting of 20 patients in original cohort of noninfectious uveitis cases underwent two additional NFC measurements 6 and 12 months after the first measurement. These 20 patients did not use any systemic medication the first NFC measurement.

### Nailfold capillaroscopic technique

A standardized NFC technique from the European Alliance of Associations for Rheumatology (EULAR) study group on Microcirculation in Rheumatic Diseases was used for the examination and classification [12, 14, 25]. Prior to the NFC examination (CapillaryScope 200 Pro, Dino-Lite), each participant was acclimatized for at least 15 min at room temperature (20–24°C). The nailfold of all fingers, except for the thumbs, was examined after applying a drop of immersion oil on the nailfold bed to improve resolution. NFC was performed by a single observer, first at low (50x) magnification in order to determine the distribution of any obvious abnormalities and to obtain an overview of the nailfold area (1–2 images were obtained). Next, four consecutive images were obtained at 200 × magnification for assessing the detailed morphology of the capillaries. The images were analyzed at a later stage by the same observer who obtained the images. Three different second observers also analyzed images for the presence of hemorrhages and to determine the capillary morphology. The intraclass correlation coefficient for the assessment of hemorrhages and abnormal morphology was 0.57–0.88; all cases with a low correlation were discussed together until consensus was reached. During the analysis, the first and second observers were both blinded with respect to the participants’ details. DinoCapture 2.0 software was used to analyze the images [26].

The following NFC parameters were assessed at 200 × magnification:

- *Capillary density*, defined as the number of capillary loops per mm in the distal row using the “90º method”, where a capillary loop was considered to be a distal loop if the apex of the capillary made an angle of ≥ 90º with the apex of its adjacent capillaries [27].
- *Density of dilated capillaries*, defined as the number of dilated capillaries with an apical limb diameter of 20–50 µm per mm [12, 14].
- *Microhemorrhages* visible as hemosiderin deposits within the distal row of the nailfold, caused by the rupture of one or more capillaries [12, 14].
- *Avascular areas*, defined as the absence of capillaries in the distal row with a minimum of 200 µm between adjacent capillaries [18].
- *Capillary morphology*, scored per capillary per image as normal, multiple crossings, tortuous, bushy, ramified, nonconvex, or bizarre shaped [12, 27]. The definitions of each capillary morphology are shown in Table 1. Capillaries with busy, ramified, nonconvex, and bizarre morphologies were also classified as having an abnormal morphology [12, 14, 25]. The percentage of each morphology type was calculated by dividing the total number of capillaries of that morphology type by the total number of capillaries assessed per image.

**Table 1:** Definitions of the various types of capillary morphologies [12, 13, 26–28]

Capillary morphology	Definition
Normal	Capillaries with a hairpin shape or a once crossing shape, on the condition that the apex is convex
Multiple crossings	The capillary limbs cross at least twice
Tortuous	Curled capillary limb without crossing of the limb
Ramified	Branched capillary limb, like a tree without leaves
Bushy	Capillary loop with limbs originating from small and multiple buds instead of branches
Nonconvex	The form of the apex is not curved like the exterior of a circle or sphere (convex)
Bizarre	Atypical morphology, distinct from the other described morphologies

tortuous, ramified, bushy, and bizarre capillaries are considered “abnormal”

**Fig. 1:**
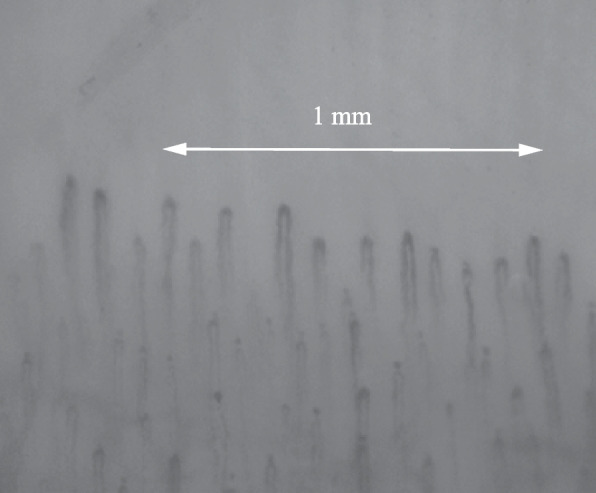
Normal nailfold capillaroscopy image of a 17-year-old female control, with a capillary density of 10 capillaries per linear mm. Note the absence of dilated capillaries, hemorrhages, and avascular areas, with normal capillary morphology

To obtain the NFC parameters at the participant level, mean values were calculated, except for the presence of microhemorrhages, in which the presence of a microhemorrhage in at least one image was considered positive for that participant. We also assessed the quality of the images and excluded any participant in which fewer than 25% of the images were deemed eligible for assessment [14]. Figure 1 shows a representative NFC image of a pediatric control.

### Statistical analysis

All statistical analyses were performed using the R software package version 4.2.2. The intraclass correlation coefficient between observers was calculated for the assessment of capillary morphology. Descriptive statistics are used to report demographics and to describe the NFC parameters per group. Except where indicated otherwise, summary data are represented as the mean and standard deviation (for continuous variables) or percentage (for categorical variables). To compare NFC parameters between groups, we performed a multivariable regression analysis, adjusted for age and sex. Differences were considered significant at *P* < 0.05, and all tests were 2-tailed.

## Results

Initially, a total of 154 patients and controls were examined with NFC, after which 10 participants (9 patients and 1 control) were excluded based on an insufficient number of assessable images. Thus, a total of 144 participants (119 patients and 25 controls) were included in our analysis. The demographics and clinical features of these groups are summarized in Table 2. Twelve of the 38 patients diagnosed with JIA-associated uveitis received systemic treatment for their uveitis. In the other patients systemic treatment was indicated for both JIA and uveitis.

**Table 2:** Participant characteristics of patients with pediatric uveitis and pediatric controls

Demographics	Uveitis	Pediatric controls	*P-*value
Number of subjects	119	25	
Male sex, *n* (%)	52 (44)	13 (52)	0.54
Mean age in years (SD)	13.7 (3)	9.1 (4)	**< 0.001**
Type of uveitis, *n* (%)		NA	
Anterior	55 (46)		
Intermediate	21 (18)		
Panuveitis	43 (36)		
Associated systemic disease, *n* (%)		NA	
None	62 (52)		
JIA	38 (32)		
Presumed TINU syndrome	13 (11)		
Other^a^	6 (5)		
Disease duration of uveitis in years	4.7 (4)	NA	
Type of JIA, *n* (%)			
Monoarthritis	2	NA	
Oligoarthritis	28		
Polyarthritis	8		
ANA seropositive, *n* (%)	60 (50)	NA	
Systemic treatment, *n* (%)			
None	25 (21)	25 (100)	
Corticosteroids	16 (13)	0	
Methotrexate	45 (38)	0	
Mycophenolate mofetil	45 (38)	0	
Adalimumab	36 (30)	0	
Infliximab	3 (3)	0	
Tocilizumab	10 (8)	0	
Other^b^	4 (3)	0	

*ANA* antinuclear antibody, *DMARD* disease-modifying antirheumatic drug, *JIA* juvenile idiopathic arthritis, *NA* not applicable, *SD* standard deviation, *TINU* tubulo-interstitial nephritis and uveitis

^a^Vogt-Koyanagi-Harada Disease (*n* = 3), psoriasis vulgaris (*n* = 2), or Blau syndrome (*n* = 1)

^b^ Azathioprine (*n* = 1), Golimumab (*n* = 1), Leflunamide (*n* = 1), Tofacitinib (*n* = 1)

### Comparison between cases and controls

We found significant differences between the patient group and the control group with respect to the number of dilated capillaries per mm, the prevalence of ramified capillaries, and the mean percentage of ramified capillaries (defined as the number of ramified capillaries divided by the total number of capillaries). Analysis of patients without an associated systemic disease (*n* = 61) showed similar results (data not shown). No other NFC parameters differed significantly between the two groups, even when we combined the four abnormal morphologies (busy, ramified, nonconvex, and bizarre) into one category (Table 3).

**Table 3:** Summary of nailfold capillaroscopy parameters in the noninfectious uveitis group and pediatric controls

Nailfold capillaroscopic parameters	Uveitis, *n* = 119	Pediatric controls, *n* = 25	*P–*value^a^
Capillary density, mean (SD)	7.2 (0.7)	7.5 (0.7)	0.05
Capillary density < 7/mm, *n* (%)	50 (42)	5 (20)	0.07
Dilated capillaries/mm, median (IQR)	0.2 (0–0.4)	0 (0–0.1)	**0.04**
Microhemorrhages,* n* (%)	17 (17)	8 (32)	0.97
Avascular areas, median (IQR)	0.3 (0.2–0.4)	0.2 (0.1–0.3)	0.20
**Capillary morphology**^**b**^			
Multiple crossings, mean % (SD)	5 (4)	2 (3)	0.06
Tortuous capillaries, mean % (SD)	7 (6)	6 (6)	0.68
Bushy capillaries, mean % (SD)	1 (1)	1 (1)	0.23
Ramified capillaries, mean % (SD)	2 (2)	1 (1)	**0.02**
Nonconvex, mean % (SD)	3 (2)	4 (2)	0.16
Bizarre capillaries, mean % (SD)	2 (3)	1 (2)	0.17
Abnormal morphology^c^, mean % (SD)	5 (4)	5 (4)	0.79

*IQR* interquartile range, *SD* standard deviation

^a^ Calculated using a multivariate regression analysis adjusted for age at NFC examination and sex

^b^ The percentage of each morphology type was calculated by dividing the sum of the number of capillaries with that morphology type by the total number of capillaries assessed per image

^c^ Abnormal morphology includes bushy, ramified, nonconvex, and bizarre shaped capillaries

### Uveitis subtypes

**Fig. 2:**
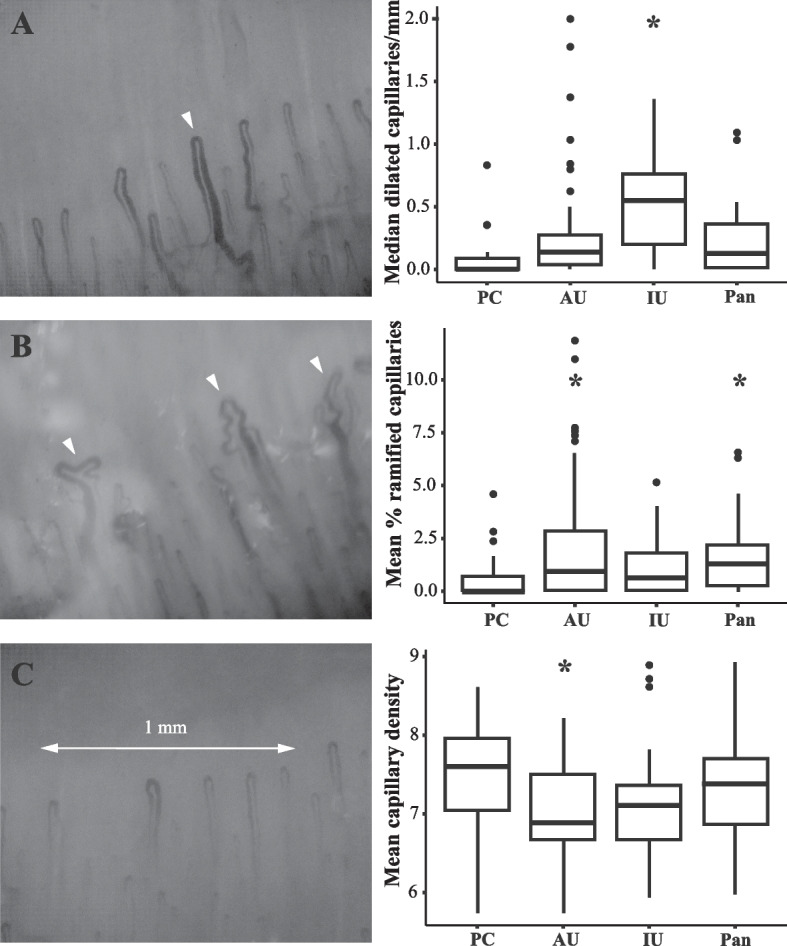
Nailfold capillaroscopy findings for pediatric controls (PC; *n* = 25), anterior uveitis cases (AU, *n* = 55), intermediate uveitis cases (IU, *n* = 21), panuveitis cases (Pan, *n* = 43). A. NFC image of a 16-year-old boy diagnosed with panuveitis showing a dilated capillary (left, arrowhead) and boxplot summarizing the median number of dilations per linear mm in each subgroup (right). B. NFC image of the same 16-year-old boy diagnosed with panuveitis showing ramified capillaries (left, arrowheads) and boxplot summarizing the mean percentage of ramified capillaries in each subgroup (right). C. NFC image of a 16-year-old girl diagnosed with anterior uveitis showing low capillary density (< 7 capillaries per linear mm) (left) and boxplot summarizing mean capillary density in each subgroup (right). *P < 0.05 versus the control group, determined using linear regression analysis adjusted for age and sex

Next, we analyzed the NFC parameters in the pediatric controls and in each of the three specific uveitis subtypes (anterior uveitis, intermediate uveitis, and panuveitis); the results are summarized in Fig. 2. We found that mean capillary density was significantly lower in the patients with anterior uveitis (7.0 ± 0.7) compared to the control group (7.5 ± 0.7, *P* = 0.008). Moreover, 54% of the patients with anterior uveitis (30/55 cases) had a capillary density < 7/mm, compared to 20% (5/25) of the pediatric controls (*P* = 0.01). We also found that the patients with intermediate uveitis had a significantly higher number of dilated capillaries per mm compared to the pediatric controls (*P* < 0.001). Finally, the mean percentage of ramified capillaries was higher in the patients with anterior uveitis and in the patients with panuveitis compared to the pediatric controls.

We also found differences in certain NFC parameters when we compared the 55 patients with anterior uveitis with the intermediate uveitis and panuveitis patients combined (*n* = 64). Specifically, the patients with anterior uveitis had a lower mean capillary density (7.0/mm vs. 7.3/mm, respectively, *P* = 0.008), a higher percentage of abnormally shaped capillaries (6% vs. 4%, respectively, *P* = 0.02), and a higher percentage of tortuous capillaries (7% vs. 4%, respectively, *P* = 0.02); all other parameters were similar between these two subgroups.

In addition, we found no difference between the 38 patients with JIA-associated uveitis and the 16 patients with idiopathic chronic anterior uveitis (data not shown). When comparing the presence of anterior chamber flare in patients with anterior uveitis, we found a higher percentage of bushy capillaries in patients without the presence of flare compared to patients with anterior chamber flare (*n* = 17, 0.9% vs. *n* = 38, 0.3%; *P* = 0.04). The other NFC-parameters were not significantly different. Also no difference was found within the different types of JIA.

Additionally, we compared ANA-positive patients (*n* = 60) to ANA-negative patients (*n* = 59), and found that ANA-positive patients had a significantly lower capillary density (7.0 vs. 7.3, respectively, *P* = 0.02) and more microhemorrhages (*P* = 0.04) compared to ANA-negative patients.

We also assessed the association between NFC parameters and inflammatory vascular involvement of the retina based on FA findings in 47 patients. We found that capillary density was significantly higher in the patients with papillitis (7.4/mm) compared to the patients without papillitis (7.1/mm, *P* = 0.04); however, this difference was no longer significant after we corrected for uveitis subtype. In contrast, we found no significant association between CME, retinal vasculitis and/or retinal capillary leakage on FA and any of the NFC parameters.

Finally, we compared patients with a capillary density < 7/mm (*n* = 50) to patients with a capillary density ≥ 7/mm (*n* = 69). We found that patients with a capillary density < 7/mm had a longer duration of uveitis, and more patients were treated with biological agents for adequate disease control compared to patients with a capillary density ≥ 7/mm when adjusting for age, sex, and duration of uveitis. We did not found an association with disease activity, and use of medication.

### Follow-up NFC data

A total of 20 patients in the uveitis group underwent two follow-up NFC measurements 6 and 12 months after the first measurement. We found no significant difference in NFC parameters between the first NFC measurement and either of the two follow-up measurements (data not shown). Interestingly, however, we found that the percentage of microhemorrhages decreased—albeit not significantly—between the initial NFC measurement (55%) and the 6-month and 12-month measurements (33% and 35%, respectively).

## Discussion

In this study, we report significant differences in NFC parameters between children with noninfectious uveitis and pediatric controls, suggesting involvement of the systemic microcirculation in pediatric uveitis. Our results also suggest that the systemic microcirculation is affected even in idiopathic cases of uveitis that present without an identified systemic condition. Previous studies have shown that several inflammatory indicators such as the neutrophil/lymphocyte ratio, interleukins, and the platelet/lymphocyte ratio are elevated in the serum of patients with noninfectious uveitis [28–39]. These increased indicators are associated with both the activity and the severity of uveitis, likely implying activation of the systemic immune system (i.e., an inflammatory index). Our results support the notion that uveitis is not exclusively an intraocular inflammation but can also indicate systemic involvement. Although the long-term consequences of these changes in the microcirculation are currently unknown, our findings underscore the complexity of this potentially sight-threatening condition in children and provides new insights for developing diagnostic and prognostic biomarkers to monitor the microcirculation.

A previous cross-sectional case–control study in adults with uveitis found differences in NFC assessment, with a higher number of dilated capillaries and lower capillary density in the patient group [18]. Although we found no difference in capillary density between our entire pediatric uveitis group and the control group, we did find a significantly lower capillary density in the patients with anterior uveitis. This difference between studies might be due—at least in part—to differences between adult patients and pediatric patients and/or the slightly higher percentage of anterior uveitis patients in the previous study in adults (54%) [18] compared to our study (46%).

Recently, Melsens et al*.* reported that healthy children have a similar capillary density as adults and that the same cut-off value used for adults (≥ 7 capillaries per linear mm) can also be used in children [14]. Although not statistically significant, we found that 42% of the children with uveitis in our study had a capillary density < 7/mm compared to 20% of pediatric controls; this difference may be attributed in part to the fact that nearly half of the patients in our study had anterior uveitis, as anterior uveitis is often accompanied by a systemic disease such as JIA. However, the presence of JIA cannot fully explain this finding, as we found no difference between JIA-associated uveitis and idiopathic chronic anterior uveitis (data not shown). In addition, these two subtypes of uveitis are considered to be clinically identical, as they share genetic risk alleles and cannot be distinguished based solely on ophthalmological features [40, 41]. Moreover, recent studies in children with JIA found no changes in NFC parameters between patients with JIA (but for whom the presence of uveitis was unknown) and controls, indicating that arthritis might not be the cause of the changes in the microcirculation [14, 42]. Despite no association was found between lower capillary density and disease activity in this study, we do believe that a lower capillary density might be associated with a worse disease control. To reach an adequate disease control these patients needed the addition of a biological agent in contrary to patients with higher capillary density.

Interestingly, we found that the patients with intermediate uveitis had the highest number of dilated capillaries per mm. Intermediate uveitis is often accompanied by signs of inflammatory involvement of the retina such as intraocular perivasculitis, vasculitis, and periphlebitis. In contrast to other studies in adults [18, 43], we found no apparent correlation between abnormalities in the retinal and nailfold microcirculation.

While vessels in the anterior uveal tract may potentially be affected especially in anterior uveitis, there is currently no practical method available to directly visualize them in clinical practice. However, the measurement of the anterior chamber flare could potentially serve as a clinical representation of this inflammatory process and the consequent breakdown of the blood-aqueous barrier as a result of it. We found no association between anterior chamber flare and NFC-parameters, with the exception of bushy capillaries. However, the found differences were very small, which might question the clinical relevance of this finding. The ciliary body might also be affected in anterior uveitis. However, it is difficult to investigate possible abnormalities regarding the vasculature with the current methods of examination.

Nailfold capillaroscopic changes are extensively described and investigated in several rheumatic diseases such as systemic sclerosis, SLE, and dermatomyositis [11–14]. Interestingly, in literature, ocular manifestations, including abnormalities in the microcirculation of the retina and choroid, are described in these rheumatic diseases [44–50]. Several of these studies use optical coherence tomography angiography (OCT-A) as a non-invasive tool to assess the retinal and choroidal microcirculation [44, 45, 47, 49, 50]. Jakhar et al. revealed that patients with systemic sclerosis with retinal disease have more severe NFC changes [51]. Studies investigating a possible association between NFC changes and retinal/choroidal microcirculatory changes are currently lacking. Therefore, future research addressing this topic might provide valuable information regarding the interaction between the microcirculation of the nailfold and the retina/choroid.

One strength of our study is that we included a relatively large cohort of children with uveitis. Furthermore, we assessed the NFC images using a standardized protocol based on the international consensus definitions established by the EULAR Study Group on Microcirculation in Rheumatic Diseases [12, 25]; these definitions were used recently in a standardized assessment of children [14]. In addition, the majority of the images was assessed by two observers who were blinded with respect to the participant’s details.

Despite these strengths, our study also has some limitations that warrant discussion. First, the pediatric control group was not age-matched to the patient group, resulting in a significant difference in age between the two groups; however, we corrected for both age and sex in of our analyses. Second, data whether patients with JIA-associated uveitis were receiving immunosuppressive drugs primarily for arthritis or for uveitis was not always clearly available in this study. Third, our prospective study did not show any apparent change in NFC findings, even after one year; however, this may have been too short of a follow-up period to detect changes, as a previous study involving patients with systemic sclerosis found that the median time to progression of the NFC pattern (i.e., the time after which 50% of patients did progress) was nearly four times as long as our follow-up period [52]. Although not statistically significant, we did observe a decrease in microhemorrhages in our prospective study. Whether this is due to effects of treatment is currently unclear due to the small number of patients. The clinical repercussions of the findings in this study are currently unknown and are yet to be determined in future studies. Longitudinal studies with repeated NFC measurements for a longer follow-up period are therefore needed in order to determine whether NFC findings have prognostic value with respect to predicting the course of disease severity in noninfectious uveitis. Furthermore, it would be interesting to investigate whether NFC findings can be used to predict disease relapse, the need for additional systemic treatment, and/or treatment response in patients with pediatric uveitis. On the other hand, future studies investigating the underlying pathophysiology are also needed in order to identify biomarkers specific to uveitis-associated inflammation and/or neoangeogenesis. Such studies will likely provide valuable information regarding the underlying disease mechanisms and may provide simple prognostic indicators that can be used to guide the precision care of patients with noninfectious uveitis.

## Conclusions

Pediatric uveitis can present with changes in the systemic microcirculation, with specific differences based on the subtype of uveitis. Our results suggest that noninfectious pediatric uveitis is not always limited to intraocular inflammation, but may also include systemic inflammation. The changes observed in the systemic microcirculation do not appear to be correlated with retinal vascular inflammation involvement in our clinically heterogenous cohort. Thus, whether capillary changes reflect vascular involvement in specific uveitis subtypes—for example, intermediate uveitis—warrants further investigation. Nevertheless, our findings provide important new insights into this severe, potentially sight-threatening condition in children and provide input for designing both prospective studies and translational studies.

### Supplementary Information

**Additional file 1**. Supplementary Material.

## Data Availability

The datasets used and/or analyzed during the current study are available from the corresponding author on reasonable request.

## Abbreviations

ANA: Antinuclear antibody
CME: Cystoid macular edema
EULAR: European Alliance of Associations for Rheumatology
FA: Fluorescein angiography
IQR: Interquartile range
JIA: Juvenile idiopathic arthritis
NFC: Nailfold capillaroscopy
OCT: Optical coherence tomography
SD: Standard deviation
