# Supplementary material for: Involvement of the systemic microcirculation in pediatric uveitis
Source: Pediatr Rheumatol Online J. 2023 Oct 2;21:109. doi: 10.1186/s12969-023-00896-7 (PMC10544362; doi:10.1186/s12969-023-00896-7)
Supplement: Supplementary file 1 — Additional file 1. Supplementary Material. [file 12969_2023_896_MOESM1_ESM.docx]

**Supplementary Material**

**Screening for associated systemic diseases in pediatric uveitis patients**

The performed tests for screening might be adjusted in individual cases based on clinical indication. The routine screening includes serology, urinary and virology testing, and chest-X-ray.

**Laboratory serology tests**Sodium, potassium, erythrocyte sedimentation rate (ESR), C-reactive protein (CRP), complete blood count including blood differential, antinuclear antibody serology, human leukocyte antigen (HLA)-B27, angiotensin-converting enzyme (ACE), calcium, albumin.

**Urinary tests**Beta2-mircoglobulin, creatinine, protein/creatinine ratio.

**Virology tests**Treponema pallidum, QuantiFERON, human immunodeficiency virus (HIV).

**Chest-X-ray**
